# Effects of Dietary Chitosan on Growth, Antioxidant Capacity, Non-Specific Immunity, and Intestinal Health of the Mud Crab, *Scylla paramamosain*

**DOI:** 10.3390/ani16060987

**Published:** 2026-03-22

**Authors:** Xiaojing Chen, E Lin, Kai Zhang, Shuangli Hao

**Affiliations:** 1 College of Marine Sciences, South China Agricultural University, Guangzhou 510642, China; 20233140006@stu.scau.edu.cn (X.C.); 20243140044@stu.scau.edu.cn (E.L.); 20243140093@stu.scau.edu.cn (K.Z.); 2 Fishery Research Institute, Nansha-South China Agricultural University, Guangzhou 511464, China

**Keywords:** chitosan, growth performance, antioxidant capacity, non-specific immunity, intestinal health, *Scylla paramamosain*

## Abstract

This study investigated the effects of dietary chitosan supplementation at levels of 0, 0.5, 1, 2, 4, and 8 g/kg on the mud crab (*Scylla paramamosain*) during an eight-week feeding trial. Results revealed significant improvements in final body weight (FBW), weight gain rate (WGR), and specific growth rate (SGR) as chitosan levels increased, with the 2 g/kg group showing the strongest effects (*p* < 0.05). Chitosan supplementation also elevated crude protein content in the whole body, muscle, and hepatopancreas (*p* < 0.05). Moreover, it notably reduced malondialdehyde (MDA) levels in hemolymph and hepatopancreas while enhancing antioxidant enzyme activity (*p* < 0.05). Chitosan at doses of 1–4 g/kg also significantly increased the activity of non-specific immune enzymes in hemolymph (*p* < 0.05). Additionally, chitosan enhanced mRNA expression of immune-related genes in the hepatopancreas (*p* < 0.05). According to gut microbiota study, 2 g/kg chitosan raised the relative abundance of advantageous gut bacteria and modulated microbial composition. In conclusion, appropriate dietary chitosan supplementation successfully stimulates mud crab growth, strengthens immunological and antioxidant systems, and improves intestinal health.

## Simple Summary

As the mud crab aquaculture industry grows, improving its health and growth performance has become a critical focus. Chitosan, a bioactive polysaccharide, holds the potential to modulate the physiological functions of aquatic organisms. This study explored the impact of supplementing the diet of mud crabs with different concentrations of chitosan. The results showed that appropriate supplementation levels simultaneously enhanced growth performance, improved antioxidant capacity and immunity, and modulated gut microbiota structure. Overall, the findings confirm the potential of chitosan as a functional feed additive in mud crab farming, providing a dosage reference and theoretical support for its practical application.

## 1. Introduction

The mud crab, *Scylla paramamosain*, is a warm-water, euryhaline, carnivorous marine crustacean classified taxonomically within the phylum Arthropoda, class Crustacea, order Decapoda, suborder Brachyura, family Portunidae, and genus *Scylla*. As an economically important mariculture species in China, its production scale and output have consistently increased owing to continuous advancements in artificial propagation, larval rearing, grow-out technologies, and aquaculture infrastructure. As recorded in the 2025 edition of the China Fisheries Statistical Yearbook, the national mariculture area for this species reached 24,602 hectares in 2024, with an output of 162,349 tons, reflecting a 3.4% increase compared to the production in 2023 [1].

With the robust development of the mud crab industry, research on formulated feeds has advanced considerably [2,3]. Such feeds can be tailored to meet specific nutritional needs, which improves feed efficiency while reducing water pollution from residual feed and lowering the incidence of disease [4]. However, the current mud crab farming still relies predominantly on frozen raw biogenic feeds. The expansion of farming scale has led to substantial inputs of such frozen feeds, resulting in deterioration of the aquaculture environment and frequent occurrences of viral [5], bacterial [6], and parasitic diseases [7]. Additionally, environmental factors, such as temperature, dissolved oxygen, ammonia nitrogen, pH, and heavy metals, also contribute to high mortality in mud crabs [8]. These issues collectively pose significant challenges to the mud crab aquaculture industry’s long-term development.

Antibiotics have been commonly incorporated into feed to prevent diseases by eliminating pathogenic bacteria, to promote growth, and to enhance immunity in farmed animals [9]. However, long-term and intensive use of antibiotics contributes to multiple issues, including the development of bacterial resistance, disruption of intestinal microbiota balance, drug residue accumulation, and environmental pollution [10]. Additionally, husbandry practices such as medicated baths and vaccination further increase labor and economic costs in aquaculture. To foster the sustainable development of aquaculture, it is essential to develop safe and effective feed additives that enhance immunity and disease resistance, while promoting the growth of farmed species.

Polysaccharide feed additives, including chitin, yeast polysaccharides, and algal polysaccharides, have been extensively studied in aquaculture species [11,12,13]. Chitin, a crucial structural polysaccharide, is present in the cell walls of fungi and algae, as well as in the exoskeletons of arthropods such as shrimp, crabs, and insects. In terms of natural abundance by biomass, chitin is defined as the second most prevalent polysaccharide worldwide, following cellulose [14]. Chitosan is produced through chemical deacetylation of chitin and is therefore also referred to as deacetylated chitin [15]. This natural polymer exhibits biocompatibility, non-toxicity, edibility, and biodegradability [16]. As a feed additive, chitosan demonstrates broad biological activities including antimicrobial and antioxidant effects, along with physiological regulatory functions such as cholesterol reduction and immune modulation [17]. These biological functions are typically assessed by measuring a set of physiological parameters. These parameters mainly include antioxidant enzymes (such as superoxide dismutase and catalase), immune-related enzymes (such as lysozyme, acid phosphatase, and alkaline phosphatase), and the lipid peroxidation product malondialdehyde [18]. These indicators reflect the physiological response of aquatic animals to dietary interventions. Chitosan supplementation has been demonstrated to enhance golden pompano (*Trachinotus ovatus*) growth performance [19]. The serum lysozyme activity in Nile tilapia (*Oreochromis niloticus*) was significantly elevated in response to chitosan-supplemented diets, with levels notably higher compared to the control diets [20]. Additionally, it was demonstrated by Li et al. that dietary chitosan exhibited antibacterial efficacy against *Aeromonas hydrophila* in Yellow catfish (*Pelteobagrus fulvidraco*) [21]. Together, these findings indicate that chitosan supplementation in feed supports the healthy development of farmed aquatic animals.

However, the impact of chitosan on the growth, body composition, non-specific immunity, and gut microbiota of this species remains unclear. Thus, this study investigated how various dietary chitosan levels affected mud crabs’ growth performance, body composition, antioxidant capacity, nonspecific immunological response, and intestinal function. It is anticipated that the findings would offer a theoretical basis for developing formulated feeds and applying chitosan as a functional feed addition in sustainable mud crab aquaculture.

## 2. Materials and Methods

### 2.1. Experimental Crab and Feeding Management

Guangzhou Nansha Farm provided the crabs for the trial. The crabs were acclimatized in culture buckets and fed a basal diet for 7 days. Thereafter, 270 healthy crabs (~43 g average weight) were randomly allocated into 18 buckets, with 15 crabs per bucket and three replicates per treatment. Each group was fed their respective experimental diet twice daily for 8 weeks. The health status of the crabs was checked every day during the trial period. The following conditions were maintained: temperature of 26–29 °C, pH of 7.6–8.0, dissolved oxygen ≥ 5.0 mg/L, salinity of 15–16‰, nitrite < 0.1 mg/L, and total ammonia nitrogen < 0.4 mg/L.

### 2.2. Experimental Diet

To prepare the experimental diets, chitosan (purity ≥ 90%, CAS: 9012-76-4, Sangon Biotech) was supplemented into a basal diet (composition shown in Table 1) at levels of 0, 0.5, 1.0, 2.0, 4.0, and 8.0 g/kg diet, designated as groups SO, SA, SB, SC, SD, and SE, respectively. All basal ingredients were ground and sieved, and due to the low inclusion levels, chitosan was premixed with a small amount of basal feed (5–10%) before being gradually blended with the remaining ingredients. After adding fish oil and water, the mixture was extruded into 3.5 mm pellets, dried at 60 °C in a multi-purpose dryer, and stored at −20 °C until use.

### 2.3. Sample Collection

After the 8-week feeding study, crabs were placed on ice to be anesthetized. Three crabs per replicate had intestinal, hepatopancreatic, and hemolymph samples taken at random. A sterile syringe was used to remove approximately 0.5 mL of hemolymph from the basal region of the fourth pereiopod, which was then deposited in a centrifuge tube with 0.5 mL of anticoagulant. The material was centrifuged for 20 min at 12,000 r/min and 4 °C. For enzyme activity tests, the resultant supernatant was kept at −80 °C. To assess immunological and antioxidant parameters, fresh hepatopancreas specimens were immediately preserved in liquid nitrogen and subsequently stored at −80 °C. Intestines were dissected and placed in 5 mL centrifuge tubes that had already been filled with 3 mL of 4% paraformaldehyde solution for histological sectioning. For each replicate, two additional crabs were sampled. Their intestines containing luminal contents were collected into sterile centrifuge tubes, immediately flash-frozen using liquid nitrogen, and maintained at −80 °C for subsequent gut microbiota analysis.

### 2.4. Determination of Growth Performance

The growth performance indices included survival rate (SR), weight gain rate (WGR), specific growth rate (SGR), and molting frequency (MF). These parameters were calculated as follows:

SR (%) = (final number/initial number) × 100.

WGR (%) = [(final crab weight (g) − initial crab weight (g))/initial weight (g)] × 100.

SGR (%/d) = [(Ln final weight − Ln initial weight)/Duration of experiment days] × 100.

MF = 2 × molting number/(initial number + final number).

### 2.5. Proximate Composition Analysis

To evaluate moisture content, feed and tissue samples were dried at 105 °C until a constant weight was achieved. The crude protein concentration was determined using the Kjeldahl method. Crude lipid content was measured through Soxhlet extraction. Crude ash content was calculated by burning samples at 550 °C in a muffle furnace. The composition of dry matter (DM), crude protein (CP), ether extract (EE), and ash in whole crabs, hepatopancreas, and muscle tissues was analyzed following AOAC standard methods [22].

### 2.6. Biochemical Index Analysis

Antioxidant parameters and immune-related enzyme activity in the hepatopancreas and hemolymph were measured using commercial kits from Nanjing Jiancheng Bioengineering Institute (Nanjing, China). The measured indicators included acid phosphatase (ACP), alkaline phosphatase (AKP), malondialdehyde (MDA), total superoxide dismutase (T-SOD), catalase (CAT), and total antioxidant capacity (T-AOC). Lysozyme (LZM) and phenol oxidase (PO) activities in hemolymph were assayed using a kit from Grace Bio (Suzhou, China). All operations and calculations strictly followed the manufacturers’ instructions.

### 2.7. Real-Time Quantitative PCR Analysis

Total RNA was extracted from crab hepatopancreas using RNAiso reagent (Vazyme, Nanjing, China), and its concentration was measured with a microspectrophotometer. First-strand cDNA was synthesized from the isolated RNA using the PrimeScript™ RT Master Mix kit (Takara, Beijing, China) following the manufacturer’s instructions. Quantitative real-time PCR (RT-qPCR) was performed on a QuantStudio thermal cycler with ChamQ SYBR qPCR Master Mix Kit, using gene-specific primers (Table 2) synthesized by Sangon Biotech (Shanghai, China). The relative expression levels of target genes (*STAT*, *crustin*, *proPO*, *JAK*, *relish*, and *Toll-like receptor*) were calculated using the 2^−ΔΔCT^ method, with *GAPDH* as the internal reference gene.

### 2.8. Intestinal Histology

A graded ethanol series was used to dehydrate the intestinal tissue after it had been fixed in 4% paraformaldehyde. Tissues were then made translucent using xylene before being implanted in paraffin. The paraffin blocks were sectioned into 5 μm thick slices. The sections were then stained with hematoxylin and eosin (H&E) and mounted with neutral resin to create permanent histological slides. Finally, the morphological structure of the intestinal tissue was assessed via light microscopy, and photographs were acquired using SlideViewer image analysis software (v2.5.0.143918).

### 2.9. Intestinal Microflora Analysis

Intestinal samples were sent to Shanghai Meiji Biotechnology Co., Ltd. (Shanghai, China) for processing and sequencing. After obtaining DNA from intestinal microbiota, the universal primers 341F and 806R were utilized to amplify the DNA using PCR. Representative sequences of Amplicon Sequence Variants (ASVs) and relative abundance data were obtained through sequencing performed on MiSeq and Novaseq platforms. Using these ASV data, multiple statistical and visual assessments were conducted. These analyses included ASV taxonomic classification, microbial community diversity evaluation, and community composition profiling.

### 2.10. Statistical Analysis

GraphPad Prism 9.0.0 and SPSS 25 were used to handle and analyze the data. The mean ± standard deviation (SD) is used to display all experimental data. One-way ANOVA and Duncan’s test were used to compare differences between the control and treatment groups. Polynomial regression analysis was used to estimate the optimal dietary chitosan level. Statistical significance was set at *p* < 0.05.

## 3. Results

### 3.1. Growth Performance

**Figure 1:**
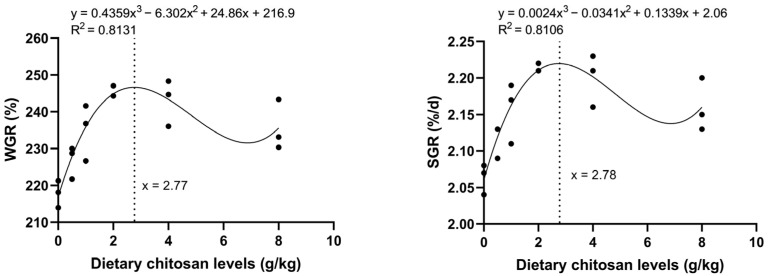
Polynomial regression for the relationship between WGR and SGR against chitosan levels in *S. paramamosain*.

After 8 weeks of feeding, dietary chitosan at 1–8 g/kg significantly improved FBW, WGR, and SGR in crabs (Table 3). The SC group showed the highest values for these parameters (*p* < 0.05). SR ranged from 46.67% to 60.00% with no significant differences among groups (*p* > 0.05). Compared with the SO group, MF was significantly higher in the SC and SD groups (*p* < 0.05). A cubic regression analysis was conducted to evaluate the effects of dietary chitosan levels. The results showed that the optimal levels for crabs were 2.77 g/kg and 2.78 g/kg. These levels supported the maximum WGR and SGR, respectively (Figure 1).

### 3.2. Proximate Composition in Whole Body, Muscle and Hepatopancreas

Dietary chitosan significantly affected the proximate composition of mud crabs (Table 4). The crude protein content in the whole body, muscle, and hepatopancreas increased significantly in the SB, SC, and SD groups (*p* < 0.05). The crude lipid content in the hepatopancreas showed an initial increase followed by a decrease, with a significant increase in the SC group and a significant decrease in the SE group compared to the SO group (*p* < 0.05). A similar trend was observed in the whole body. The crude ash content in the whole body, muscle, and hepatopancreas also exhibited an initial increase followed by a decrease, with the highest values observed in the SC group (*p* < 0.05).

### 3.3. Immune Enzyme Activity in Hemolymph

**Figure 2:**
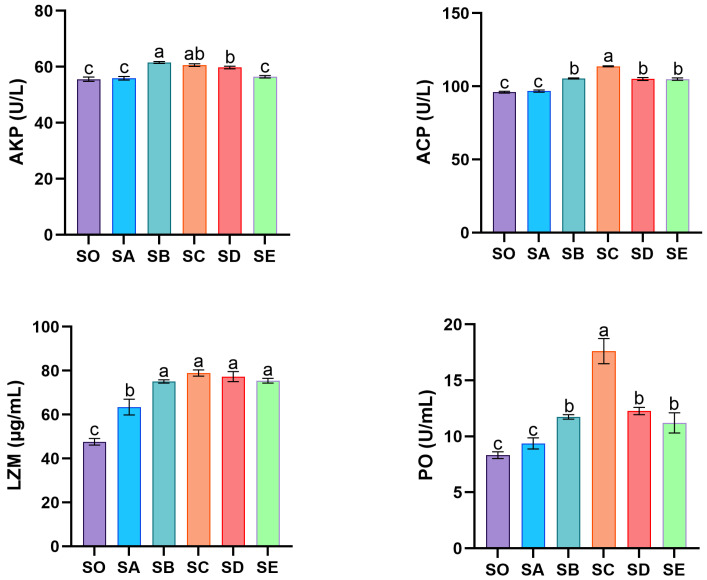
Effects of chitosan on the immune indexes of haemolymph in *S. paramamosain*. Significant differences exist between bars labeled with different superscripts (*p* < 0.05). AKP, Alkaline phosphatase; ACP, Acid phosphatase; LZM, Lysozyme; PO, Phenoloxidase.

The activities of non-specific immune-related enzymes in the hemolymph of mud crabs are shown in Figure 2. AKP activity was significantly elevated in the SB, SC, and SD groups compared to the remaining three groups (*p* < 0.05). The SB, SC, SD, and SE groups all had considerably higher levels of ACP activity than the SO group, while the SC group exhibited the highest level (*p* < 0.05). The SA (lowest chitosan concentration) and SO groups did not significantly differ in ACP activity. PO activity followed a trend similar to that of ACP, with the SC group again demonstrating the most pronounced activity (*p* < 0.05). After feeding with varying chitosan concentrations, LZM activity was notably elevated in each treatment group relative to the control (*p* < 0.05).

### 3.4. Antioxidant Enzyme Activities in Hemolymph and Hepatopancreas

**Figure 3:**
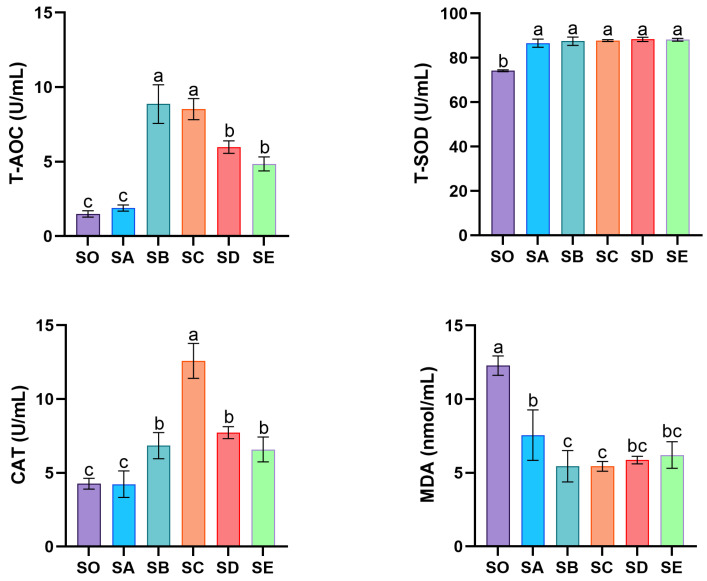
Effects of dietary chitosan on the antioxidant status of haemolymph in *S. paramamosain*. Significant differences exist between bars labeled with different superscripts (*p* < 0.05).

The effect of dietary chitosan supplementation on the antioxidant capacity of mud crab hemolymph is shown in Figure 3. The activity of T-AOC and CAT exhibited a pattern of initial rise and subsequent reduction. T-AOC activity was considerably higher in the SB and SC groups than in the other groups, whereas the lowest activity was detected in the SO and SA groups (*p* < 0.05). In the SC group, CAT activity was highest (*p* < 0.05). Supplementation with varied chitosan concentrations increased T-SOD activity and lowered MDA content in crab hemolymph compared to the SO group (*p* < 0.05).

**Figure 4:**
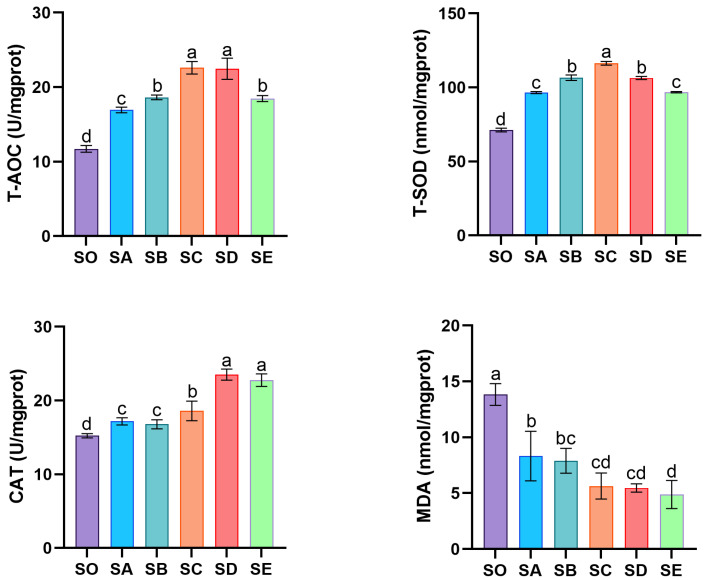
Effects of chitosan on the antioxidant status of hepatopancreas in *S. paramamosain*. Significant differences exist between bars labeled with different superscripts (*p* < 0.05).

Dietary chitosan supplementation significantly affected the antioxidant capacity in the hepatopancreas of mud crabs (Figure 4). T-AOC, T-SOD, and CAT activities were enhanced, while MDA content decreased with increasing chitosan levels. T-AOC and T-SOD activities first increased and then decreased, with the highest values observed in the SC group, followed by the SB and SD groups (*p* < 0.05). CAT activity was significantly higher in all chitosan-supplemented groups compared with the SO group (*p* < 0.05). MDA content showed a decreasing trend with increasing chitosan levels, and all treatment groups had significantly lower MDA content than the SO group (*p* < 0.05).

### 3.5. The mRNA Expression of Non-Specific Immune-Related Genes in Hepatopancreas

**Figure 5:**
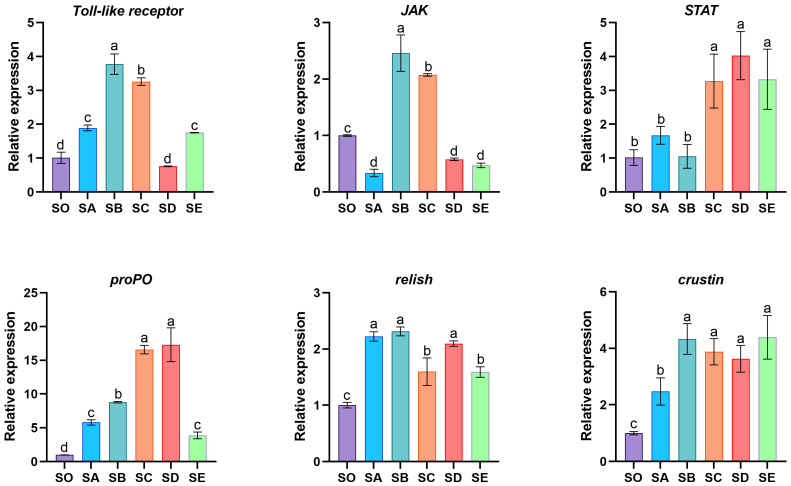
Effects of chitosan on immune-related gene expression of hepatopancreas in *S. paramamosain*. Significant differences exist between bars labeled with different superscripts (*p* < 0.05).

The relative mRNA expression levels of non-specific immune-related genes *Toll-like receptor*, *JAK*, *STAT*, *proPO*, *relish*, and *crustin* in the hepatopancreas of mud crabs are presented in Figure 5. In the SB and SC groups, *Toll-like receptor* and *JAK* mRNA expression was considerably raised (*p* < 0.05). Relative to the SO, SA, and SB groups, the SC, SD, and SE groups had considerably higher *STAT* gene expression levels (*p* < 0.05). With increasing chitosan concentration in the diet, mRNA expression of *proPO* in the hepatopancreas first increased and subsequently decreased. Specifically, the SC and SD groups exhibited considerably higher *proPO* expression (*p* < 0.05). All treatment groups had significantly greater expression of *relish* and *crustin* mRNA than the SO group (*p* < 0.05).

### 3.6. Intestinal Morphology

**Figure 6:**
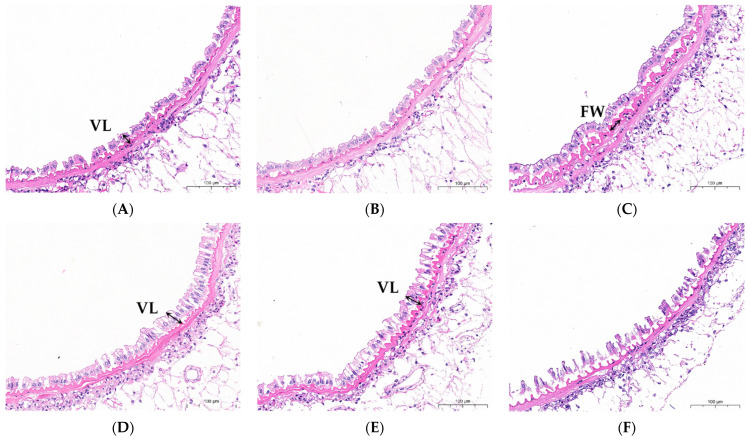
Effects of dietary chitosan supplementation at 0 (**A**), 0.5 (**B**), 1 (**C**), 2 (**D**), 4 (**E**), and 8 (**F**) g/kg on the intestinal histology of *S. paramamosain*. VL: villus length, FW: Fold width. Scale bar = 100 μm.

As shown in Figure 6, the SB, SC, and SD groups exhibited tighter cellular organization in intestinal tissues compared with the SO group. However, the intestinal tissue in the SE group was loosely arranged, and the structure of the intestine was disordered. As shown in Table 5, the fold width (FW) in the SB group was significantly increased compared with the SO group, while the villus length (VL) in the SC and SD groups was significantly longer (*p* < 0.05). No significant differences were observed between the SA and SO groups (*p* > 0.05).

### 3.7. Intestinal Microflora

The SC group showed the most favorable overall results, followed by the SB and SD groups, according to the thorough evaluation of growth performance, antioxidant capacity, and immune response in mud crabs. Accordingly, the SC group, SB group, and the SO group were selected for gut microbiota analysis using 16S rRNA gene sequencing.

**Figure 7:**
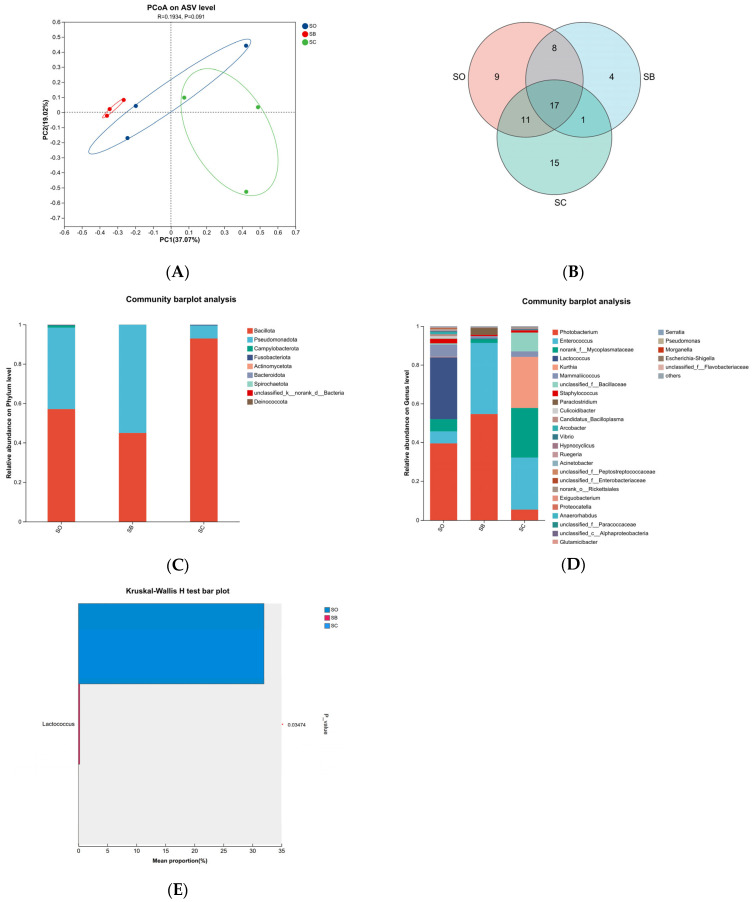
Changes in the diversity and richness of intestinal microbial of *S. paramamosain* based on ASV level. Principal coordinate analysis (PCoA) of microbial communities between samples (**A**). Venn diagram of shared and specific microbiota communities among groups (**B**). Relative abundances of the dominant bacterial phylum (**C**) and genus (**D**) from different chitosan diets treatments. Kruskal–Wallis H test bar plot showing significantly different genera among groups (**E**).

Alpha diversity indices are summarized in Table 6. There were no discernible variations among groups in terms of community richness (Ace index, Chao index, Sobs index) or community diversity (Shannon index, Simpson index) (*p* > 0.05). Principal coordinate analysis (PCoA) was used for beta diversity analysis in order to display sample differences (Figure 7A). The gut microbiota composition of the SC group supplemented with chitosan was similar to that of the SO control group. However, the intestinal microbiota composition of crabs in the SB group, which received a chitosan-supplemented diet, showed changes compared to that of the SO group. This result indicates certain differences in species composition between the two groups. Permutational multivariate analysis of variance (PERMANOVA) was further conducted to quantify the contribution of the grouping factor to the variation in microbial communities (Table 7). The grouping factor explained 32.19% of the community variation (R^2^ = 0.3219), although this result was not statistically significant (*p* = 0.134).

A total of 119 representative amplicon sequence variants (ASVs) were identified across all groups (Figure 7B). The SO, SB, and SC groups contained 9, 4, and 15 unique ASVs, respectively, with 17 ASVs shared among all three. At the phylum level (Figure 7C), Bacillota and Pseudomonadota dominated the gut microbiota in all groups. Following dietary chitosan treatment, the SC group had a greater relative abundance of Bacillota in contract to the SO group. *Photobacterium* and *Lactococcus* in the SO group; *Photobacterium* and *Enterococcus* in the SB group; and *Enterococcus*, *Kurthia*, and *norank_f_Mycoplasmataceae* in the SC group were the predominant taxa at the genus level (Figure 7D). *Photobacterium* was highly abundant in both the SO and SB groups, while the most prevalent genus observed within the SC group was *Enterococcus*. Notably, the SB and SC groups had considerably greater relative abundances of *Enterococcus* than the SO group. Multi-comparison analysis (Figure 7E) showed that *Lactococcus* was the dominant biomarker in the control group. Its abundance decreased significantly after the chitosan treatment (*p* < 0.05).

## 4. Discussion

The growth-promoting effects of chitosan, primarily attributed to enhanced immune stimulation and improved intestinal health, have been established in various animal species. In this study, cubic regression analysis estimated that the dietary chitosan levels for optimal WGR and SGR of mud crab were 2.78 g/kg and 2.77 g/kg, respectively. Compared with the control group, the final body weight, weight gain rate, and specific growth rate in the SB, SC, SD, and SE groups were significantly increased. Among the chitosan-supplemented groups, MF was significantly elevated only in groups SC and SD. However, the low-chitosan (SA) group did not show any growth-promoting effects. The results showed that dietary chitosan supplementation at 1–8 g/kg effectively promoted the growth of mud crab. The optimal supplementation level was estimated to be approximately 2.77–2.78 g/kg. This outcome aligns with numerous previous studies. For example, tilapia supplemented with 0.4% chitosan had higher WGR and SGR [20]. Chen et al. discovered that adding chitosan to diets significantly improved WGR in loaches, indicating that chitosan promotes loach growth [24]. Further studies indicate that diets enriched with 4–8 g/kg of chitosan considerably enhanced FBW, SGR, and WGR in *Trachinotus ovatus* [19]. Comparable findings have also been observed with chitosan nanoparticles in Nile tilapia [25]. It should be noted that the overall survival rate in this experiment was low. This was primarily attributed to the intraspecific cannibalism behavior of crabs during the molting period. Due to the limitations of the culture conditions, some soft-shell crabs lacked effective shelter and were easily attacked and killed by other crabs. However, the key point is that there was no significant difference in survival rate among the treatment groups. This indicates that the mortality was evenly distributed across all groups and did not introduce systematic bias into the inter-group comparison of the core indicators. Therefore, the effects of chitosan on mud crabs observed in this study are considered reliable.

In this study, dietary supplementation with an appropriate level of chitosan (1–4 g/kg) significantly increased the crude protein content in the whole body, muscle, and hepatopancreas of mud crabs, indicating that chitosan promotes protein deposition. A similar finding was reported by Li et al. [21], who observed a significant increase in whole-body crude protein content in juvenile yellow catfish fed diets supplemented with 5–10 g/kg chitosan for eight weeks. The underlying mechanism may involve the regulation of amino acid metabolism and the reduction in transdeamination, leading to a protein-sparing effect that enhances protein deposition [26]. As the central organ for lipid metabolism in crustaceans, the hepatopancreas is responsible for lipid digestion, synthesis, and storage, and is particularly sensitive to changes in dietary nutrients and additives [27]. In the present study, dietary chitosan at 1 and 2 g/kg significantly increased hepatopancreatic crude lipid content, whereas 8 g/kg chitosan significantly decreased it. This result is consistent with the findings of Niu et al. [28] in Pacific white shrimp, where low-dose chitosan supplementation increased crude lipid content and high-dose supplementation decreased it. Considering the concurrent increase in crude protein content, it is speculated that low to moderate doses of chitosan promote overall nutrient deposition, and the increase in lipid content may represent an energy reserve adjustment to support rapid growth. In addition, the antioxidant activity of chitosan may also contribute to lipid retention. Wang et al. [29] demonstrated that chitosan reduces serum lipid peroxidation levels and minimizes lipid oxidative loss, thereby facilitating lipid accumulation in the body. When the chitosan dose exceeds a certain threshold, its role in binding dietary lipids in the intestine becomes predominant, resulting in reduced lipid absorption and deposition [30]. In this study, the crude ash content in the whole body, muscle, and hepatopancreas reached its maximum in the SC group. Ash content reflects the overall level of mineral elements in the body, and its variation suggests that chitosan may influence the absorption and deposition of minerals.

Under normal physiological settings, cells continuously generate reactive oxygen species (ROS). However, when ROS levels generated by oxidative stress exceed a certain threshold, biomolecules become damaged [31]. Consequently, organisms activate antioxidant defense systems to eliminate excess ROS, thereby reducing oxidative damage and preventing pathological changes [32]. The evaluation of antioxidant capacity and related biomarkers (T-AOC, SOD, CAT, and MDA) effectively reflects the organisms oxidative stress status [33]. The antioxidant system’s first line of defense is made up of natural antioxidant enzymes called SOD and CAT. Superoxide anion radicals are converted by SOD into hydrogen peroxide (H_2_O_2_), which is then further broken down by CAT into H_2_O and O_2_. This process determines whether ROS are effectively removed [34]. When the body encounters harmful stimuli, excessive cellular ROS production causes lipid membrane peroxidation and the creation of MDA [35]. MDA is a crucial marker for determining the extent of oxidative damage since it is a byproduct of lipid peroxidation. In this study, dietary chitosan notably increased T-AOC, T-SOD, and CAT activity in mud crab hemolymph and hepatopancreas while decreasing MDA content. These outcomes are compatible with the findings of Thilagar et al., who demonstrated that chitosan dramatically increased SOD, T-AOC, and CAT activities in tilapia following lead stress [36]. Similarly, after being fed diets enriched with chitosan nanoparticles for 8 weeks, fish receiving 1 and 2 g/kg exhibited lower MDA levels and higher SOD, CAT, and GPx activity than those fed 0 and 0.5 g/kg [37]. Therefore, chitosan’s antioxidant effect can be attributed to its capacity to eliminate ROS in cells by enhancing T-AOC, T-SOD, and CAT activities, thereby mitigating oxidative stress damage to the organism [38].

The immunity of crustaceans is mainly accomplished by some non-specific immune enzymes and other immune factors. LZM, a hydrolase discovered in aquatic animal serum and phagocytes, breaks down the β-1,4 glycosidic bond between N-acetylmuramic acid and N-acetylglucosamine in Gram-positive bacteria’s peptidoglycan cell wall. This process disrupts the cell wall, releases intracellular contents, and induces bacterial lysis [39]. PO resists pathogen invasion, enhances hemocyte phagocytosis, and mediates bacterial agglutination and clearance processes [40]. LZM and PO activities are considered key indicators of aquatic animal immune status. Studies indicate that feeding loach diets containing 1, 5, and 10 g/kg chitosan significantly increased both LZM and PO activities in contrast to the control group [24]. Similar outcomes were found in the present study. The hemolymph of mud crabs exhibited a notable increase in LZM activity following the inclusion of chitosan in their diet, and the 2 g/kg group had a much greater PO activity than the other groups. Upon stimulation by foreign substances, lysosomal membranes rupture to release hydrolases. As typical hydrolases, ACP and AKP play a role in pollutant detoxification and removal processes [41]. Their activities typically reflect an animal’s immune status [42]. This study showed that ACP and AKP activities in mud crab hemolymph were considerably increased by diets enriched with 1, 2, and 4 g/kg of chitosan. In Nile tilapia, dietary chitosan alleviates cadmium stress by enhancing non-specific immunity through increased serum ACP, AKP, and lactate dehydrogenase (LDH) activity [43]. These findings suggest that dietary chitosan supplementation may enhance aquatic animals’ resistance to pathogen infection.

The Toll signaling pathway, the IMD signaling pathway, and the JAK/STAT signaling pathway are recognized as the primary signaling pathways regulating the innate immune response of crustaceans [44]. Toll-like receptors (TLRs) are important membrane recognition receptors that activate NF-κB expression through interaction with MyD88 [45]. This process subsequently modulates the production of key antimicrobial peptides, such as *crustin*, *lysozyme*, and anti-lipopolysaccharide factor (*ALF*), thereby eliminating pathogens within crustaceans [46]. Relish, an NF-κB homolog in invertebrates, participates in regulating inflammatory responses and apoptosis [47]. JAK is a protein tyrosine kinase whose mediated phosphorylation activates signal transduction and transcription activator (STAT) proteins. STAT, as a transcription factor, initiates the expression of genes associated with antibacterial and antiviral immunity [48,49]. The proOP system is also a crucial component of crustacean innate immunity, forming an immune defense mechanism through a series of proteases [50]. In this study, feeding mud crabs a diet containing 2 g/kg chitosan significantly upregulated the gene expression levels of *Toll-like receptor*, *relish*, *crustin*, *JAK*, *STAT*, and *proPO* in the hepatopancreas. Similarly, the addition of bamboo leaf flavonoids [51], mannooligosaccharides [52], and inulin [53] significantly increased gene expression levels of *TLRs*, *STAT*, *proPO*, *crustin*, and *ALF* in shrimp and crab. The results above indicate that dietary chitosan up-regulates the expression of several immune-related genes in the hepatopancreas of mud crabs. Based on the known functions of these genes in the Toll pathway, the JAK/STAT pathway, and the proPO system, it is suggested that chitosan may enhance immune function by influencing these immune pathways. However, it should be noted that this study only reveals a correlation between gene expression and health improvement. The direct causal mechanisms still require further investigation.

Animal digestion, absorption, and growth are all significantly impacted by the intestine, an essential organ for nutrition metabolism and absorption. Generally, taller, wider, and denser intestinal villi and folds signify higher efficiency of nutrient absorption [54,55]. The results of this investigation showed that the supplemental diet of 1–4 g/kg of chitosan increased the villus density, villus length and fold width in the intestine of mud crabs. Similar results have been published in related research. Zake et al. discovered that dietary supplementation of 1.5–6 g/kg chitosan increased both villus height and width in juvenile stellate sturgeon (*Acipenser stellatus*) [56]. Similarly, Salam et al. [57] and Kamali Najafabad et al. [58] found that supplementation with 1 g/kg chitosan significantly increased intestinal villus length in juvenile *Barbonymus gonionotus* and caspian kutum (*Rutilus frisii kutum* Kamenskii, 1901), respectively. These studies indicate that dietary supplementation of appropriate levels of chitosan can improve intestinal morphology. Chitosan can enhance nutrient absorption by increasing the intestinal absorptive surface area via enlarged villus dimensions (height and width) and increased density.

Intestinal health significantly influences animal growth. Maintaining a stable balance of intestinal microorganisms assists the host in resisting pathogenic infections and preventing disease [59]. The Ace, Chao1, Shannon, and Simpson indices in this study did not significantly differ between the groups according to alpha diversity analysis. The results indicate that dietary chitosan did not alter the species richness or diversity of the intestinal microbial community in mud crabs. This suggests that chitosan did not cause major changes in the number of species. It also did not disrupt the overall ecological structure of the microbial community [60]. PERMANOVA analysis showed that the grouping factor did not have a statistically significant effect on microbial community structure. However, this factor explained 32.19% of the community variation, indicating a potentially large effect size. This relatively high explanation suggests that chitosan may have some potential impact on the intestinal microbial community.

Venn diagram analysis showed that three groups shared 17 ASVs. These formed a relatively stable core microbiota. The number of unique ASVs varied among treatments. This indicated that chitosan addition altered the composition of the intestinal microbiota. Among all groups, the SC group (2.0 g/kg) had the highest number of unique ASVs. This suggested that this dose had the most significant regulatory effect on the intestinal microbiota.

Zhang proposed that the Proteobacteria, Firmicutes and Mollicutes constitute the dominant shared microbial communities in the intestines of decapods such as shrimp and crabs [61]. Hong et al. also identified the phyla Firmicutes, Proteobacteria, Bacteroidetes, and Actinobacteria as potential dominant phyla in crustacean gut microbiota [62]. Thus, Firmicutes, Proteobacteria, Bacteroidetes, and Actinobacteria may be the phylum-level dominant gut microbiota in shrimp and crabs. This study suggests that the dominating phyla in mud crabs’ intestinal microbiota are primarily Bacillota (formerly Firmicutes) and Pseudomonadota (formerly Proteobacteria), consistent with previous research findings. Pseudomonadota includes numerous opportunistic pathogens. Their overproliferation can disrupt gut microbial community structure, leading to intestinal diseases [63]. Bacillota contains denitrifying bacteria, including various probiotics like *Enterococcus*, *Lactobacillus*, and *Bacillus*. These bacteria synthesize diverse digestive enzymes to facilitate food digestion, stimulate innate immune responses to secrete antimicrobial peptides, and strengthen the intestinal barrier to prevent pathogen adhesion, ultimately achieving the goal of preventing intestinal infections [64]. Dietary treatment with 2 g/kg chitosan significantly increased Bacillota relative abundance while reducing Pseudomonadota relative abundance in comparison with the control group. These findings imply that supplementing feed with 2 g/kg chitosan may enhance intestinal immunity in mud crabs and reduce the incidence of intestinal diseases. Similar studies have shown that adding 500, 1000, or 2000 mg/kg of Astragalus polysaccharides to feed increases the relative abundance of the Firmicutes in Chinese mitten crabs [65], while supplementing feed with 320 mg/kg of Guava leaves extracts reduces the relative abundance of the Proteobacteria in mud crabs [66].

*Photobacterium* constitute the predominant genus in the control group. Belonging to the Pseudomonadota, they are potential pathogenic microorganisms found on the body surface and in the intestines of aquatic animals. *Photobacterium damselae* subsp. *damselae* is the primary pathogenic species within the genus, capable of inducing photobacteriosis in mud crabs, which negatively affects crab growth [67]. In the group treated with 2 g/kg chitosan, the relative abundance of *Photobacterium* was considerably lower compared to the control group. This suggests that 2 g/kg chitosan may enhance the host’s resistance against pathogenic bacteria. The genera *Lactococcus* and *Enterococcus* belong to the phylum Bacillota. The genus *Lactococcus* exhibits functional diversity across different hosts. In the intestines of *Macrobrachium rosenbergii*, *Lactococcus* exhibited the highest abundance, with *Lactococcus garvieae* playing a crucial role as a dominant species in shrimp growth and development, but most species within the *Lactococcus* are pathogenic to fish [68]. *Enterococcus* has been reported as a probiotic. Specifically, *Enterococcus faecalis* has been shown to enhance nonspecific immunity in *Litopenaeus vannamei*, promote nutrient digestion and absorption, and improve intestinal health [69]. In this investigation, meals supplemented with 1 and 2 g/kg chitosan had an increased relative abundance of *Enterococcus* than the control group. This suggests chitosan may improve intestinal function by increasing *Enterococcus* abundance, thereby potentially benefiting mud crab growth. Meanwhile, the relative abundance of *Lactococcus* showed a decreasing trend with increasing chitosan supplementation. These results indicate that dietary chitosan can optimize the intestinal microbial community structure of mud crabs. While maintaining the overall ecological balance of the gut microbiota, chitosan suppressed potentially harmful bacteria and enriched potentially beneficial bacteria, thereby improving the microbial community structure.

## 5. Conclusions

Feeding mud crabs a diet supplemented with chitosan at levels of 1–8 g/kg for 8 weeks maintains their favorable growth performance and antioxidant capacity. Based on cubic regression analysis of WGR and SGR, the optimal supplementation level was estimated to be approximately 2.77–2.78 g/kg. Notably, dietary chitosan levels of 1–4 g/kg significantly enhance non-specific immunity. Furthermore, a 2 g/kg chitosan level reduced pathogenic bacteria abundance and increased beneficial microbial abundance in the gut, thereby improving intestinal health. In conclusion, based on the parameters evaluated in the present study, the optimum dietary chitosan inclusion level for mud crabs is estimated to be 2.0–2.8 g/kg. This study establishes the foundation for the potential application of chitosan in mud crab feed formulations.

## Figures and Tables

**Table 1:** Ingredients and proximate compositions of the experimental diets.

Ingredient (g/kg in Dry Matter)	Content (g/kg Diet)					
	SO	SA	SB	SC	SD	SE
Fish meal	410.0	410.0	410.0	410.0	410.0	410.0
Casein	180.0	180.0	180.0	180.0	180.0	180.0
Fish oil	80.0	80.0	80.0	80.0	80.0	80.0
α-starch	200.0	200.0	200.0	200.0	200.0	200.0
Vitamin premix	30.0	30.0	30.0	30.0	30.0	30.0
Mineral premix	20.0	20.0	20.0	20.0	20.0	20.0
Cholesterol	8.0	8.0	8.0	8.0	8.0	8.0
Soybean lecithin	10.0	10.0	10.0	10.0	10.0	10.0
Ca(H_2_PO_4_)_2_	15.0	15.0	15.0	15.0	15.0	15.0
Choline chloride	7.0	7.0	7.0	7.0	7.0	7.0
Sodium alginate	10.0	10.0	10.0	10.0	10.0	10.0
Squid paste	10.0	10.0	10.0	10.0	10.0	10.0
Microcrystalline Cellulose	20.0	19.5	19.0	18.0	16.0	12.0
Chitosan	0.0	0.5	1.0	2.0	4.0	8.0
Proximate composition (% dry matter)						
Moisture	10.99	10.81	11.04	10.95	10.83	11.11
Crude protein	41.86	41.65	42.55	42.92	41.94	41.78
Crude lipid	12.91	11.79	12.75	12.90	12.75	12.93
Crude ash	8.99	9.06	9.24	10.18	9.57	9.77

Note: Vitamin premix supplied the following per kg of the diet: calcium pantothenate, 2.5 g/kg; inositol, 10 g/kg; nicotinamide, 4 g/kg; biotin, 8 mg/kg; vitamin C phosphate, 30 g/kg; vitamin A, 4 × 10^5^ IU/kg; vitamin D, 2 × 10^5^ IU/kg; vitamin E, 3 g/kg; vitamin K, 1 g/kg; vitamin B1, 0.5 g/kg; vitamin B2, 1.5 g/kg; vitamin B6, 0.8 g/kg; vitamin B12, 2 mg/kg; folic acid, 0.25 g/kg. Mineral premix supplied the following per kilogram of diet: 3.75 g/kg per kilogram of feed; copper, 1.5 g/kg; zinc, 5 g/kg; magnesium, 6 g/kg; cobalt, 80 mg/kg; selenium, 1.8 mg/kg; iodine, 3 mg/kg.

**Table 2:** Sequences of primers used for RT-qPCR.

Name	Primer Sequence, Sense/Anti-Sense	Genbank/Reference
*GAPDH*	ACCTCACCAACTCCAACAC	JX268543.1
	CATTCACAGCCACAACCT	
*JAK*	TTGCGGTGCTTCAGTTTC	KC711048.1
	TCAAGATTGGCGAGGGTA	
*STAT*	GACTTCACTAACTTCAGCCTCG	KC711050.1
	GAGCTGAGTCTGTCTTAATGTTATCC	
*proPO*	ATGAAAGAGGAGTGGAGATG	FJ160768.1
	GTGATGGATGAGGAGGTG	
*Toll-like receptor*	TGTTGCCAGAGCAGAAGGT	LT835105.1
	TTCCGTGAATGAACGAAGG	
*crustin*	AAAGCACTATGCGCAAAGAAAACT	[23]
	CACCTTCTGGTAATAGATTATT	
*relish*	AGTGGAACAGTGGTCCAGCTG	
	CACCACCACTTCACAAATC	

Note: *JAK*, *Janus kinase*; *STAT*, *Signal Transducer and Activator of Transcription*; *proPO*, *Prophenoloxidase*.

**Table 3:** Effects of dietary chitosan on growth performance of *S. paramamosain*.

Growth Index	SO	SA	SB	SC	SD	SE
IBW (g)	43.04 ± 0.47	42.22 ± 0.78	42.74 ± 0.59	43.39 ± 0.39	43.26 ± 0.59	42.96 ± 0.81
FBW (g)	136.75 ± 0.59 ^c^	137.97 ± 1.25 ^c^	143.15 ± 1.34 ^b^	150.20 ± 1.26 ^a^	148.38 ± 0.83 ^a^	144.13 ± 0.85 ^b^
WGR (%)	217.76 ± 3.67 ^d^	226.83 ± 4.47 ^cd^	235.03 ± 7.65 ^bc^	246.15 ± 1.59 ^a^	243.02 ± 6.30 ^ab^	235.61 ± 6.85 ^bc^
SGR (%)	2.06 ± 0.02 ^d^	2.11 ± 0.02 ^cd^	2.16 ± 0.04 ^bc^	2.22 ± 0.01 ^a^	2.20 ± 0.03 ^ab^	2.16 ± 0.04 ^bc^
SR (%)	46.67 ± 6.67	57.78 ± 3.85	60 ± 6.67	60 ± 11.54	55.56 ± 3.85	46.67 ± 6.67
MF	0.32 ± 0.09 ^c^	0.33 ± 0.06 ^bc^	0.34 ± 0.08 ^bc^	0.47 ± 0.05 ^a^	0.44 ± 0.04 ^ab^	0.4 ± 0.06 ^abc^

Note: Data are reported as the mean ± SD of three replicates (*n* = 3). Different superscripts letters in the row show a significant difference between treatments (*p* < 0.05).

**Table 4:** Proximate compositions (wet weight basis) of whole body, muscle and hepatopancreas of *S. paramamosain* fed different levels of dietary chitosan for 8 weeks.

Parameters	SO	SA	SB	SC	SD	SE
**Whole body**						
Moisture (%)	67.56 ± 0.44 ^a^	67.58 ± 0.37 ^a^	62.09 ± 0.15 ^c^	62.13 ± 0.16 ^c^	64.35 ± 0.61 ^b^	66.96 ± 0.34 ^a^
Crude protein (%)	13.05 ± 0.26 ^c^	12.71 ± 0.15 ^c^	16.01 ± 0.24 ^a^	15.84 ± 0.24 ^a^	14.15 ± 0.28 ^b^	13.09 ± 0.28 ^c^
Crude fat (%)	4.31 ± 0.19 ^bc^	4.44 ± 0.20 ^ab^	4.78 ± 0.20 ^a^	4.62 ± 0.17 ^ab^	4.28 ± 0.23 ^bc^	4.02 ± 0.10 ^c^
Crude ash (%)	5.60 ± 0.16 ^bc^	5.65 ± 0.30 ^bc^	6.12 ± 0.35 ^ab^	6.38 ± 0.36 ^a^	6.36 ± 0.32 ^a^	5.45 ± 0.30 ^c^
**Muscle**						
Moisture (%)	75.75 ± 0.58 ^a^	75.24 ± 0.66 ^a^	73.35 ± 0.25 ^b^	72.26 ± 0.62 ^c^	73.21 ± 0.38 ^b^	74.90 ± 0.17 ^a^
Crude protein (%)	17.05 ± 0.12 ^d^	17.87 ± 0.23 ^c^	19.54 ± 0.15 ^b^	19.98 ± 0.17 ^a^	19.82 ± 0.07 ^a^	18.07 ± 0.04 ^c^
Crude fat (%)	2.27 ± 0.22 ^ab^	2.00 ± 0.37 ^b^	2.19 ± 0.23 ^ab^	2.29 ± 0.38 ^ab^	2.57 ± 0.15 ^a^	2.47 ± 0.11 ^ab^
Crude ash (%)	1.68 ± 0.07 ^b^	1.80 ± 0.13 ^ab^	1.82 ± 0.15 ^ab^	1.92 ± 0.10 ^a^	1.83 ± 0.13 ^ab^	1.88 ± 0.12 ^ab^
**Hepatopancreas**						
Moisture (%)	40.17 ± 0.41 ^ab^	39.72 ± 0.37 ^abc^	35.95 ± 0.24 ^c^	36.02 ± 0.22 ^c^	37.04 ± 0.15 ^bc^	42.83 ± 4.84 ^a^
Crude protein (%)	12.61 ± 0.34 ^c^	13.64 ± 0.32 ^b^	15.67 ± 0.39 ^a^	15.14 ± 0.42 ^a^	14.99 ± 0.44 ^a^	13.00 ± 0.48 ^bc^
Crude fat (%)	35.75 ± 0.48 ^b^	35.95 ± 0.43 ^b^	36.31 ± 0.35 ^b^	37.92 ± 0.31 ^a^	36.20 ± 0.63 ^b^	33.69 ± 0.52 ^c^
Crude ash (%)	4.11 ± 0.52 ^b^	4.76 ± 0.11 ^ab^	4.45 ± 0.38 ^ab^	5.00 ± 0.40 ^a^	4.77 ± 0.27 ^ab^	4.42 ± 0.28 ^ab^

Note: Data is presented as the mean ± SD of three replicates (*n* = 3). Significant differences between treatments are indicated by different superscript letters in the row (*p* < 0.05).

**Table 5:** Effects of chitosan on the intestinal tissue structure of *S. paramamosain*.

Growth Index	SO	SA	SB	SC	SD	SE
Villus length (μm)	16.03 ± 0.60 ^c^	15.83 ± 0.96 ^c^	17.77 ± 1.44 ^c^	30.83 ± 1.33 ^a^	30.13 ± 1.88 ^a^	21.07 ± 1.74 ^b^
Fold width (μm)	6.00 ± 0.46 ^cd^	6.17 ± 0.35 ^cd^	23.67 ± 1.00 ^a^	6.90 ± 0.20 ^c^	14.20 ± 1.37 ^b^	4.83 ± 0.25 ^d^

Note: Data is presented as the mean ± SD of three replicates (*n* = 3). Significant differences between treatments are indicated by different superscript letters in the row (*p* < 0.05).

**Table 6:** Effects of dietary chitosan on intestinal microbiome alpha diversity in *S. paramamosain*.

**Alpha Name**	**Ace**	**Chao**	**Sobs**	**Shannon**	**Simpson**	**Goods Coverage**
qvalue	0.49	0.49	0.49	0.49	0.49	NaN
SO	41.00 ± 13.53	41.00 ± 13.53	41.00 ± 13.53	1.55 ± 0.92	0.43 ± 0.33	1
SB	26.33 ± 7.64	26.33 ± 7.64	26.33 ± 7.64	1.54 ± 0.26	0.33 ± 0.07	1
SC	27.67 ± 15.63	27.67 ± 15.63	27.67 ± 15.63	0.99 ± 0.31	0.55 ± 0.14	1

Note: Data are reported as the mean ± SD of three replicates (*n* = 3). No significant difference is indicated by values in the same column without superscripts (*p* > 0.05).

**Table 7:** Effects of dietary chitosan on intestinal microbiome beta diversity based on PERMANOVA.

Name	Df	SumsOfSqs	MeanSqs	F.Models	R2	Pr (>F)
group	2	0.8111	0.40555	1.42391	0.32187	0.134
Residuals	6	1.70888	0.28481	-	0.67813	-
Total	8	2.51998	-	-	1	-

Note: PERMANOVA based on Bray–Curtis distance was used to test for differences in microbial community composition among groups. The analysis was conducted with 999 permutations. No significant group-level differences were detected (*p* = 0.134).

## Data Availability

The original contributions presented in this study are included in the article. Further inquiries can be directed to the corresponding author.

## References

[1] Ministry of Agriculture and Rural Affairs, National Fisheries Technology Extension Center, China Society of Fisheries (2025). China Fishery Statistical Yearbook 2025.

[2] Wenrui Z., Hongxia L., Zun W., Xiujuan W., Yubo W., Zhongcang Q., Jie W. (2023). Effect of defatted black soldier fly larvae meal on nutritional quality of *Scyla paramamosain*. Feed. Res..

[3] Ai C., Lin Q., Li S., Wang G., Chen X. (2006). Review on nutrient requirements and formulated feed of crabs *Eriocheir sinensis*, *Scylla sp.*, Portunus trituberculatus. J. Xiamen Univ. (Nat. Sci.).

[4] Huang W., Lin X., Zhong C. (2020). Effects of soft pelleted diet on survival, growth and muscular amino acid composition of mud crab *Scylla paramamosain*. Chin. J. Fish..

[5] Xiao Hui C., Yin Hui P., Xu Jia L., Chu Chao Y., Qiong S., Zhen Ping N., Lan Fang D., Zhi Hui Y., Zhi Cheng W. (2013). The Pathogenicity of Mud Crab Reovirus (MCRV) to Green Crab *Scylla paramamosain*. Fish. Sci..

[6] Lihui S., Qian L., Rongxiang J., Feng L. (2019). Isolation and Identification of Pathogenic Bacteria from Diseased *Scylla paramamosain*. Hubei Agric. Sci..

[7] Ying Z., Yulai D., Zhen T., Guoliang W. (2015). Histopathology and ultrastructure of *Hematodiniun* sp. disease in *Scylla paramamosain*. J. Biol..

[8] Jithendran K., Poornima M., Balasubramanian C., Kulasekarapandian S. (2010). Diseases of mud crabs (*Scylla* spp.): An overview. Indian J. Fish..

[9] Yang M., Li D., Zhou M. (2020). The Research and Application Progress of Feed Antibiotic Substitute in Aquaculture. Anhui Agric. Sci. Bull..

[10] Li A., Lin M. (2025). Current research status and development trends of antibiotic alternatives in aquaculture. Acta Hydrobiol. Sin..

[11] Dawood M.A.O., Koshio S., Esteban M.Á. (2017). Beneficial roles of feed additives as immunostimulants in aquaculture: A review. Rev. Aquac..

[12] Wang E., Chen X., Liu T., Wang K. (2022). Effect of dietary *Ficus carica* polysaccharides on the growth performance, innate immune response and survival of crucian carp against *Aeromonas hydrophila* infection. Fish Shellfish Immunol..

[13] Liu W.C., Zhou S.H., Balasubramanian B., Zeng F.Y., Sun C.B., Pang H.Y. (2020). Dietary seaweed (*Enteromorpha*) polysaccharides improves growth performance involved in regulation of immune responses, intestinal morphology and microbial community in banana shrimp *Fenneropenaeus merguiensis*. Fish Shellfish Immunol..

[14] Dassanayake R.S., Acharya S., Abidi N. (2019). Biopolymer-Based Materials from Polysaccharides: Properties, Processing, Characterization and Sorption Applications. Advanced Sorption Process Applications.

[15] Abu-Elala N.M., Hossam-Elden N., Marzouk M.S., Basuini M.F.E. (2025). Chitosan for Aquaculture: Growth Promotion, Immune Modulation, Antimicrobial Activity, Bio-Carrier Utility, Water Quality Management, and Safety Considerations—A Review. Ann. Anim. Sci..

[16] Mohan K., Rajan D.K., Ganesan A.R., Divya D., Johansen J., Zhang S. (2023). Chitin, chitosan and chitooligosaccharides as potential growth promoters and immunostimulants in aquaculture: A comprehensive review. Int. J. Biol. Macromol..

[17] Kamal M., Youssef I.M., Khalil H.A., Ayoub M.A., Hashem N.M. (2023). Multifunctional Role of Chitosan in Farm Animals: A Comprehensive Review. Ann. Anim. Sci..

[18] Wang W., Xue C., Mao X. (2020). Chitosan: Structural modification, biological activity and application. Int. J. Biol. Macromol..

[19] Yu W., Yang Y., Chen H., Zhou Q., Zhang Y., Huang X., Huang Z., Li T., Zhou C., Ma Z. (2023). Effects of dietary chitosan on the growth, health status and disease resistance of golden pompano (*Trachinotus ovatus*). Carbohydr. Polym..

[20] Wu S. (2020). The growth performance, body composition and nonspecific immunity of Tilapia (*Oreochromis niloticus*) affected by chitosan. Int. J. Biol. Macromol..

[21] Li S., Li C., Wu S. (2022). Dietary chitosan modulates the growth performance, body composition and nonspecific immunity of juvenile yellow catfish (*Pelteobagrus fulvidraco*). Int. J. Biol. Macromol..

[22] White W.B. (1957). AOAC Methods of Analysis. Food Drug Cosmet. Law. J..

[23] He X., Zhang H., Zhong J., Wang J., Wu K., Wen X. (2024). Regulatory mechanism of Elovl6 in lipid metabolism, antioxidant capacity, and immune function in *Scylla paramamosain* revealed by Ap-1. Int. J. Biol. Macromol..

[24] Chen J., Chen L. (2019). Effects of chitosan-supplemented diets on the growth performance, nonspecific immunity and health of loach fish (*Misgurnus anguillicadatus*). Carbohydr. Polym..

[25] El-Naby F.S.A., Naiel M.A.E., AlSagheer A.A., Negm S.S. (2019). Dietary chitosan nanoparticles enhance the growth, production performance, and immunity in *Oreochromis niloticus*. Aquaculture.

[26] Gaspar C., Silva-Marrero J.I., Fàbregas A., Miñarro M., Ticó J.R., Baanante I.V., Metón I. (2018). Administration of chitosan-tripolyphosphate-DNA nanoparticles to knockdown glutamate dehydrogenase expression impairs transdeamination and gluconeogenesis in the liver. J. Biotechnol..

[27] Vogt G. (2019). Functional cytology of the hepatopancreas of decapod crustaceans. J. Morphol..

[28] Niu J., Liu Y.J., Lin H.Z., Mai K.S., Yang H.J., Liang G.Y., Tian L.X. (2011). Effects of dietary chitosan on growth, survival and stress tolerance of postlarval shrimp, *Litopenaeus vannamei*. Aquac. Nutr..

[29] Wang Z., Yan Y., Zhang Z., Li C., Mei L., Hou R., Liu X., Jiang H. (2024). Effect of Chitosan and Its Water-Soluble Derivatives on Antioxidant Activity. Polymers.

[30] Zhang J., Zhang R., Wang P., Wen P., Zhang W., Liu S., Ren F. (2025). Impact of chitosan on lipid digestion under simulated gastro-intestinal conditions. Food Chem. X.

[31] Bhattacharyya A., Chattopadhyay R., Mitra S., Crowe S.E. (2014). Oxidative stress: An essential factor in the pathogenesis of gastrointestinal mucosal diseases. Physiol. Rev..

[32] Voronkova Y.S., Voronkova O.S., Gorban V.A., Holoborodko K.K. (2018). Oxidative stress, reactive oxygen species, antioxidants: A review. Ecol. Noospherol..

[33] Hussein E.E., Habiba M.M., Ashry A.M., Al-Zayat A.M., Teiba I.I., Shehata A.I., Shahin S.A., El-Ratel I.T., Mzengereza K., Tembo M. (2023). Effects of dietary supplementation with organic acids mixture on growth, feed efficiency, hematobiochemical parameters, immunity, and intestinal microbiota of Gilthead seabream (*Sparus aurata*) juveniles. Aquac. Rep..

[34] Nordberg J., Arner E.S. (2001). Reactive oxygen species, antioxidants, and the mammalian thioredoxin system. Free Radic. Biol. Med..

[35] Zhang Q., Li F., Guo M., Qin M., Wang J., Yu H., Xu J., Liu Y., Tong T. (2023). Growth Performance, Antioxidant and Immunity Capacity Were Significantly Affected by Feeding Fermented Soybean Meal in Juvenile Coho Salmon (*Oncorhynchus kisutch*). Animals.

[36] Thilagar G., Samuthirapandian R. (2020). Chitosan from crustacean shell waste and its protective role against lead toxicity in *Oreochromis mossambicus*. Toxicol. Rep..

[37] Dawood M.A.O., Gewaily M.S., Soliman A.A., Shukry M., Amer A.A., Younis E.M., Abdel-Warith A.A., Van Doan H., Saad A.H., Aboubakr M. (2020). Marine-Derived Chitosan Nanoparticles Improved the Intestinal Histo-Morphometrical Features in Association with the Health and Immune Response of Grey Mullet (*Liza ramada*). Mar. Drugs.

[38] Brol J., Müller L., Prates E.C.A., de Farias B.S., Pedrosa V.F., de Almeida Pinto L.A., Cadaval T.R.S., Tesser M.B., Wasielesky W., Ventura-Lima J. (2021). Dietary chitosan supplementation in *Litopenaeus vannamei* reared in a biofloc system: Effect on antioxidant status facing saline stress. Aquaculture.

[39] Liu J., Zhou N., Fu R., Cao D., Si Y., Li A., Zhao H., Zhang Q., Yu H. (2017). The polymorphism of chicken-type lysozyme gene in Japanese flounder (*Paralichthys olivaceus*) and its association with resistance/susceptibility to Listonella anguillarum. Fish Shellfish Immunol..

[40] Amparyup P., Charoensapsri W., Tassanakajon A. (2013). Prophenoloxidase system and its role in shrimp immune responses against major pathogens. Fish Shellfish Immunol..

[41] Zhao L., Yang X., Cheng Y., Liang P., Zhang J., Hong Y., Wang C., Yang Z. (2012). Effects of Histamine on Survival and Immune Parameters of the Chinese Mitten Crab, *Eriocheir sinensis*. J. Shellfish. Res..

[42] Kurhaluk N., Tkachenko H. (2022). Effects of melatonin and metformin in preventing lysosome-induced autophagy and oxidative stress in rat models of carcinogenesis and the impact of high-fat diet. Sci. Rep..

[43] Zhang Q., Xie Y., Tang J., Meng L., Huang E., Liu D., Tong T., Liu Y., Guo Z. (2024). Effects of Dietary Chitosan on Growth Performance, Serum Biochemical Indices, Antioxidant Capacity, and Immune Response of Juvenile Tilapia (*Oreochromis niloticus*) under Cadmium Stress. Animals.

[44] Li F., Xiang J. (2013). Signaling pathways regulating innate immune responses in shrimp. Fish Shellfish Immunol..

[45] Huang Y., Chen Y.H., Wang Z., Wang W., Ren Q. (2014). Novel myeloid differentiation factor 88, EsMyD88, exhibits EsTube-binding activity in Chinese mitten crab *Eriocheir sinensis*. Dev. Comp. Immunol..

[46] Li X., Cui Z., Liu Y., Song C., Shi G. (2013). Transcriptome analysis and discovery of genes involved in immune pathways from hepatopancreas of microbial challenged mitten crab *Eriocheir sinensis*. PLoS ONE.

[47] Wang J. (2023). Effects of Yeast Culture on Growth, Immune Performance and Intestinal Health of *Eriocheir sinensis*. Ph.D. Thesis.

[48] Song X., Zhang Z., Wang S., Li H., Zuo H., Xu X., Weng S., He J., Li C. (2015). A Janus Kinase in the JAK/STAT signaling pathway from *Litopenaeus vannamei* is involved in antiviral immune response. Fish Shellfish Immunol..

[49] Li C., Li H., Chen Y., Chen Y., Wang S., Weng S.P., Xu X., He J. (2015). Activation of Vago by interferon regulatory factor (IRF) suggests an interferon system-like antiviral mechanism in shrimp. Sci. Rep..

[50] Ponprateep S., Vatanavicharn T., Lo C.F., Tassanakajon A., Rimphanitchayakit V. (2017). Alpha-2-macroglobulin is a modulator of prophenoloxidase system in pacific white shrimp *Litopenaeus vannamai*. Fish Shellfish Immunol..

[51] Zhang R., Shi X., Liu J., Jiang Y., Wu Y., Xu Y., Yang C. (2022). The effects of bamboo leaf flavonoids on growth performance, immunity, antioxidant status, and intestinal microflora of Chinese mitten crabs (*Eriocheir sinensis*). Anim. Feed Sci. Technol..

[52] Lu J., Qi C., Limbu S.M., Han F., Yang L., Wang X., Qin J.G., Chen L. (2019). Dietary mannan oligosaccharide (MOS) improves growth performance, antioxidant capacity, non-specific immunity and intestinal histology of juvenile Chinese mitten crabs (*Eriocheir sinensis*). Aquaculture.

[53] Li Y., Liu H., Dai X., Li J., Ding F. (2018). Effects of dietary inulin and mannan oligosaccharide on immune related genes expression and disease resistance of Pacific white shrimp, *Litopenaeus vannamei*. Fish Shellfish Immunol..

[54] Guan F., Shen L., Zhou X., Chen Z., Yu C., Zhang J., Yuan Y. (2021). Effects of underwater and semi-aquatic environments on gut tissue and microbiota of the mudskipper *Boleophthalmus pectinirostris*. J. Comp. Physiol. B.

[55] Su X., Ji D., Yao J., Zou Y., Yan M. (2022). Comparative Analysis of Intestinal Characteristics of Largemouth Bass (*Micropterus salmoides*) and Intestinal Flora with Different Growth Rates. Fishes.

[56] Zakeri D., Pazooki J., Mohseni M., Jamshidi S. (2024). Effect of dietary chitosan on the growth performance, intestinal histology and growth-related gene expression in stellate sturgeon (*Acipenser stellatus*) juveniles. J. Anim. Physiol. Anim. Nutr..

[57] Salam M.A., Rahman M.A., Paul S.I., Islam F., Barman A.K., Rahman Z., Shaha D.C., Rahman M.M., Islam T. (2021). Dietary chitosan promotes the growth, biochemical composition, gut microbiota, hematological parameters and internal organ morphology of juvenile *Barbonymus gonionotus*. PLoS ONE.

[58] Kamali Najafabad M., Imanpoor M.R., Taghizadeh V., Alishahi A. (2016). Effect of dietary chitosan on growth performance, hematological parameters, intestinal histology and stress resistance of Caspian kutum (*Rutilus frisii kutum* Kamenskii, 1901) fingerlings. Fish Physiol. Biochem..

[59] Michaudel C., Sokol H. (2020). The Gut Microbiota at the Service of Immunometabolism. Cell Metab..

[60] Zhang Z.L., Cao Y.L., Xu J.R., Zhang X.X., Li J.J., Li J.T., Zheng P.H., Xian J.A., Lu Y.P. (2024). Effects of dietary chitosan oligosaccharide on the growth, intestinal microbiota and immunity of juvenile red claw crayfish (*Cherax quadricarinatus*). Fish Shellfish Immunol..

[61] Ning Z. (2022). Temporal and Spatial Characteristics of Intestinal Flora of Shrimp and Crab in Anhui Section of Huaihe River.

[62] Hong Y., Huang Y., Wu S., Yang X., Dong Y., Xu D., Huang Z. (2020). Effects of imidacloprid on the oxidative stress, detoxification and gut microbiota of Chinese mitten crab, *Eriocheir sinensis*. Sci. Total Environ..

[63] Shin N.R., Whon T.W., Bae J.W. (2015). Proteobacteria: Microbial signature of dysbiosis in gut microbiota. Trends Biotechnol..

[64] Smriga S., Sandin S.A., Azam F. (2010). Abundance, diversity, and activity of microbial assemblages associated with coral reef fish guts and feces. FEMS Microbiol. Ecol..

[65] Shi X., Wu C., Ma H., Liu J., Fu C., Zhou R., Jiang Y., Zhang R. (2024). Exploring the effects of astragalus polysaccharide food supplementation on growth performance, immunity, antioxidant capacity, and intestinal microflora in Chinese mitten crabs. Aquac. Rep..

[66] Yan Y. (2023). Effects of Guava Leaf Supplementation on Growth Performance, Non-Specific Immunity and Intestinal Flora of Mud Crab *Scylla paramamosain*. Master’s Thesis.

[67] Rivas A.J., Lemos M.L., Osorio C.R. (2013). Photobacterium damselae subsp. damselae, a bacterium pathogenic for marine animals and humans. Front. Microbiol..

[68] Luo J. (2023). Intestinal Flora Structure Analysis of Different Male Phenotypes in *Macrobrachium rosenbergii*. Master’s Thesis.

[69] Yu A. (2022). Effects of Different Forms of *Enterococcus faecalis* F7 Supplementation in Feed on Growth, Immunity and Intestinal Microbiota of *Litopenaeus vannamei*. Master’s Thesis.

